# Toward a Causal Science of Early Play?

**DOI:** 10.1111/infa.70033

**Published:** 2025-07-31

**Authors:** Giulia Serino, Ori Ossmy

**Affiliations:** ^1^ Centre for Brain and Cognitive Development Birkbeck University of London London UK; ^2^ School of Psychological Sciences Birkbeck University of London London UK; ^3^ Centre for Educational Neuroscience Birkbeck University of London London UK

**Keywords:** cognitive development, developmental cascades, executive functions, infancy, motor development, naturalistic experimentation, physical cognition, play

## Abstract

Young children across the globe devote much of their early years to physically engaging with the world—stacking, climbing, scribbling, and tinkering with objects. Although this embodied play is widely believed to fuel key cognitive processes like attention, memory, and executive function, most supporting evidence remains descriptive or correlational. Here, we review findings from embodied cognition research and highlight why direct experimental manipulations—rather than observations alone—are critical to demonstrating whether and how infants’ and children’s sensorimotor engagements shape their cognitive trajectories. We discuss emerging technologies (e.g., motion capture, wearable eye‐tracking) that can assess play in natural contexts, along with the use of embodied computational models for testing the impact of altered object affordances and caregiver scaffolding. We propose designs for real‐world interventions such as rotating different types of toys, systematically modifying motor demands, and tracking outcomes in attention and problem‐solving, which can bring new causal clarity to developmental science. We argue that a causal science of play will have broad implications for early education, policy, and intervention programs that aim to transform the theory of embodied cognition into practical benefits for children's learning and development.

## 1 Introduction

Physical play is a universal feature of early childhood, with infants and young children worldwide devoting a substantial portion of their early years to active exploration, object manipulation, and imaginative interactions. This is true in both Western (Lockman and Tamis‐LeMonda 2021; Tamis‐LeMonda 2023) and non‐Western cultural contexts (Boyette 2018; Karasik et al. 2024). Whether children in industrialized settings have commercial toys such as wooden blocks, tablets, and crayons, make creative use of everyday objects like stones, kettles, or plastic bottles floating in water bins (Karasik et al. 2024), or engage with culturally specific items such as buckets, sticks, and bowls (Casey et al. 2022), they consistently transform whatever materials are at hand into playful experiences. This form of playful engagement is widely considered a key driver of early learning and development.

Indeed, from infancy onward, children's physical interactions with the world appear to provide a foundation for critical cognitive processes such as attention, memory, executive function, language, and problem‐solving (Needham and Libertus 2011; Smith et al. 2018; Tamis‐LeMonda and Masek 2023). These foundational motor and sensory experiences not only support children in discovering how objects work and how physical spaces are structured but also support the formation of neural connections underlying cognitive skills (Noble et al. 2015; Oakes and Rakison 2019; Tooley et al. 2021).

To date, however, much of our understanding of children's daily physical play in real‐world settings and its contributions to cognition has been constrained by observational or correlational research designs. Many developmental studies highlight strong links between how children physically engage with objects and the forms of learning that emerge afterward—spatial reasoning, conceptual development, categorization, language learning, and even later executive functions (Campos et al. 2000; Farran et al. 2019; Iverson 2022; Oakes 2023). Yet relatively few investigations directly manipulate children's everyday environments to pinpoint causal relationships. In other words, while researchers know children's daily physical play routines and they know sensorimotor activities are associated with advanced cognition (Lockman and Tamis‐LeMonda 2021), they rarely *systematically* alter key elements of children's play settings in homes, nurseries, or schools to observe how these changes might affect their developmental trajectories.

In this narrative review, we build on classic and recent insights into the role of physical experiences during play in early cognition. We propose that to understand *how* early cognitive demands, motor actions and bodily engagements shape young children's learning and cognition, researchers must move beyond purely descriptive accounts and embrace new experimental paradigms with a clear theoretical framework and specific hypotheses about the directionality and causality of these physical experiences, and test these hypotheses by manipulating play activities, object affordances, contexts, and social partners “in the wild.” Such paradigms should harness cutting‐edge methodologies, including automatic computer‐vision analyses of spontaneous play videos, wearable eye‐tracking devices, kinematic analyses of children's physical interactions, and neurorobotic simulations that model sensorimotor development (Golmakani et al. 2025; Koziol and Lutz 2013; Le et al. 2021; Marmeleira and Duarte Santos 2019; Needham et al. 2002; Ossmy et al. 2018; Ossmy et al. 2021; Ossmy et al. 2024).

We begin by reviewing the literature on physical play in natural settings, focusing on the importance of embodiment—how the body constrains and supports the child's exploration of the world and thereby affects cognitive outcomes (Hofsten 2009). We then highlight the gaps in causal evidence and the reasons why existing naturalistic experimentations are largely descriptive or correlational. Next, we outline potential methodological pathways for conducting direct, ecologically valid manipulations that probe whether changes to infants' and young children's everyday physical play experiences alter subsequent attention, learning, memory, and executive function. Finally, we propose how these new approaches can bridge theoretical accounts of embodied cognition with real‐life applications aimed at promoting more robust learning in infants—and, as some argue, across the lifespan (R. Wu and Strickland‐Hughes 2019).

## 2 Observing Physical Experiences: Insights and Limitations

### 2.1 Embodied Cognition in the Lab as a Foundational Lens

A fundamental premise in developmental science is that infants learn about the world in and through their bodies: They grip, reach, crawl, walk, and eventually talk, building an interwoven tapestry of sensorimotor information that informs their emerging conceptual, linguistic, and socio‐emotional capacities (Adolph et al. 2019; Cole et al. 2016; Gottwald et al. 2016; Needham and Libertus 2011; Smith 2005; Smith et al. 2018; Tamis‐LeMonda and Masek 2023). The embodied cognition perspective emphasizes that “thinking” is not a wholly abstract, detached process but is grounded in the perceptual and motor systems. Indeed, *embodiment* has been proposed as a central driving force in early development, whereby infants' bodily constraints (e.g., limited postural control, immature coordination) simultaneously limit and *shape* what they can learn (Adolph et al. 2018; Cole and Adolph 2023; Koziol and Lutz 2013; Marmeleira and Duarte Santos 2019; Rachwani et al. 2022).

At any given age, infants possess certain postural and motor control capacities that enable or limit how they can explore objects and spaces (Needham and Libertus 2011). For example, young infants who cannot sit independently often rely on caregivers to position objects within reach, whereas older infants who can crawl or walk can select objects from various locations, manipulate them in different positions, and carry them across spaces (Cole and Adolph 2023; Karasik et al. 2011; Soska and Adolph 2014). These expanded possibilities for action can fundamentally alter the child's learning experiences, including what they pay attention to and how they perceive object permanence, spatial relationships, and cause‐effect patterns (Campos et al. 2000; Iverson 2022; Tamis‐LeMonda and Masek 2023). Likewise, the development of the visual system, especially during the first year of life, fundamentally impacts what infants attend to, reflecting cascading effects of the information they can access and learn about the world around them (Johnson 2005; Oakes 2023; Rueda and Posner 2013).

Research on locomotion also demonstrates this link between motor and cognitive development in Western cultures. Around the time that infants achieve crawling or walking, they experience profound shifts in perception, attention, navigation, and social‐emotional functioning (Adolph et al. 2018; Campos et al. 2000; Hospodar and Adolph 2024; Kretch et al. 2014; Oakes and Amso 2018). Locomotion expands infants' access to diverse spatial layouts and vantage points and fosters greater engagement with objects and caregivers, facilitating richer opportunities for cognitive growth (J. E. Hoch et al. 2019; Iverson 2022; Rachwani et al. 2020). Likewise, the attainment of self‐generated mobility may trigger changes in the number of stimuli infants encounter, how they regulate their attentional resources to integrate new patterns of information, manage goal‐directed behaviors, and respond to caregiver cues—all hallmarks of emerging executive function (J. J. Hoch et al. 2024; Keller et al. 2023; Oakes and Amso 2018; Smith 2013; Smith et al. 2011; Yang et al. 2023; Yu and Smith 2013).

Other researchers showed that locomotor attainment is closely tied to spatial cognition. Kermoian and Campos (1988) reported that eight‐month‐old infants with crawling or walker experience greatly outperformed non‐crawlers on a two‐location object‐search task. In their study, infants with hands‐and‐knees crawling or walker practice “facilitated spatial search performance,” and longer locomotor experience yielded higher search scores. Belly‐crawlers (inefficient locomotion) performed like prelocomotor infants unless they progressed to hands‐knees or walker modes. Similarly, Campos et al. (2009) found that infants with spina bifida—who typically begin crawling late—showed delays in spatial tasks (manual search and following an adult's point/gaze) until after they began to crawl. In other words, delayed locomotion was accompanied by delayed spatial understanding, which caught up once infants became mobile. Anderson et al. (2013) describe this transition as a “psychological revolution,” noting that independent locomotion triggers broad gains in spatial cognition (as well as memory and perceptual‐motor coordination). Correlational studies in toddlers also support this link: one large study of 19–24‐month‐olds found that gross motor competence (both locomotion and object‐control skills) was significantly positively associated with standardized cognitive scores.

Beyond spatial effects, gross motor play reshapes infants' exploratory and social behavior in ways that could influence attention, language, and early executive function. Gustafson (1984) showed experimentally that infants with walker‐supported or independent locomotion spent more time approaching caregivers and exploring objects than non‐walkers; locomoting infants had qualitatively different exploratory/social experiences than non‐locomoting infants. Similarly, in naturalistic observation, walking infants also appear to create richer learning environments. In a full‐day home recording study, Walle and Warlaumont (2015) found that adult words, infant vocalizations, and conversational turns were positively related to vocabulary growth only for walking infants. Thus, as infants gain mobility, they not only see more of the environment but also elicit different social and linguistic input.

By preschool age, differences in fine motor dexterity shape how children engage with certain toys, which, in turn, impacts the kinds of cognitive challenges they experience (Jung et al. 2015; Kahrs et al. 2014; Lockman and Tamis‐LeMonda 2021). A shape sorter that demands careful alignment of shapes to slots may scaffold attention, working memory, and planning (Lockman et al. 2018; Örnkloo and Von Hofsten 2017; Ossmy et al. 2020). By contrast, a tablet‐based app that requires only minimal tapping or swiping might reduce the child's need to plan or coordinate multiple motor actions (Lin 2019). While both types of activities may have their own benefits, they offer distinct bodily experiences that might channel cognitive development in different ways. If motor experiences significantly shift how infants perceive and interact with the environment, then different *types* of physical play might also lead to distinct patterns of cognitive development, especially if repeated daily.

Thus, investigating play in real‐world settings enables an examination of how repeated everyday actions accumulate over time, contributing to the developmental cascade that supports the emergence of complex cognitive processes. Toddlers who spend ample time stacking blocks, sorting shapes, or manipulating puzzle pieces may hone their spatial cognition differently than those who predominantly engage with digital tablets or passively watch animated videos.

Yet, confirming these intuitions requires carefully constructed hypotheses and studies that test them by quantifying the types, frequencies, and trajectories of young children's play in natural environments—and that tease apart what aspects of embodied experiences drive changes in cognition and how.

### 2.2 Descriptive Observations: Rich Data, What About Causal Clarity?

Most evidence about the links between physical *daily* experiences and cognitive outcomes arises from correlational or descriptive designs. Researchers often observe children's natural play behaviors, measure motor skills such as object‐manipulation tendencies, and correlate these with concurrent or later cognitive scores (Farran et al. 2019; J. Williams and Holley 2013). For example, repeated attempts to crawl up and down slopes might predict subsequent improvements in problem‐solving, object permanence, or language. Similarly, a child's access to varied play materials and daily physical activities in the home environment has been linked to higher developmental quotients in a Brazilian sample (Miquelote et al. 2012).

Advances in digital and analytic methods have pushed descriptive work further. These include using digital audio‐recorders for naturalistic observations of language and parenting (d’Apice et al. 2019), leveraging big data to map behavioral development (Ehlman et al. 2023; Kosie and Lew‐Williams 2024), and employing virtual visits to assess mother‐infant interactions (Shin et al. 2021).

In the field of attention, researchers map children's moment‐to‐moment looking behavior in natural environments using portable eye‐tracking glasses (Kaplan and Yu 2024; Slone et al. 2018; Yang et al. 2023), tracking fluctuations in physiological arousal and sustained attention during social and non‐social play through electrocardiogram recordings (Bradshaw et al. 2024) and electroencephalography (Amadó et al. 2023; Perapoch Amadó et al. 2025). In addition, new approaches are being developed to extend the application of functional near‐infrared spectroscopy to parent‐infant naturalistic toy play and shed light on the neural bases of attention, particularly from early in life (Dopierala and Emberson 2023; Serino et al. 2024).

Researchers have also investigated parent‐child brain‐to‐brain synchrony using hyper‐scanning techniques (Alonso et al. 2024) and developed mobile applications for automatic emotion and attention analysis in young children (Egger et al. 2018). These methods offered new insights into early social attention (Hoehl and Markova 2018), neurodevelopmental trajectories (Rogers and Luby 2023), and the impact of environmental exposures on child development (Wass et al. 2021). However, the majority of these findings remain correlational, and establishing directionality is necessary to better understand developmental cascades.

In the motor and cognitive domains, researchers used video‐recording or neural recordings of parent–child play sessions over extended periods, capturing how objects, children's exploratory actions, and adult scaffolding co‐evolve moment by moment. For example, Karmazyn‐Raz and Smith (2023) applied network analyses to map how toddlers repeatedly engage with a few “favorite” toys, interspersed with brief forays into new toy exploration. Indeed, these studies revealed the microstructures of real‐life play, however they still do not *experimentally* manipulate which objects are present or how parents might interact with them. While the descriptive data suggest that repeated sensorimotor interactions are crucial to forming categories, practicing spatial reasoning, or developing motor dexterity, a confounding factor—like a child's temperament or a caregiver's instructional style—could explain the correlation.

Thus, descriptive and correlational approaches are critical for the understanding of naturally occurring patterns of behavior and indeed have helped delineate foundational steps in developmental trajectories (Oakes and Rakison 2019). However, such approaches are still limited in ruling out alternative explanations or fully capturing how developmental influences may vary across individuals (Oakes 2023; Oakes and Rakison 2019). For example, an infant whose parents encourage repeated block‐building might simultaneously receive richer linguistic input, greater attentional support, and social scaffolding and might also exhibit a naturally higher curiosity or persistence, any of which could shape later cognitive outcomes. Consistent with this, research has shown that during joint play, parental social interaction helps infants sustain attention on objects for longer periods of time. Sustained attention, in turn, facilitates information processing (Colombo and Cheatham 2006; Holmboe et al. 2018; Xie et al. 2018) and memory encoding (Brandes‐Aitken et al. 2019; Forest and Amso 2023) and has been linked to the emergence of self‐regulation (Brandes‐Aitken et al. 2019), inhibitory control (Reck and Hund 2011), and effortful control (Frick and Baumeler 2017; Rothbart et al. 2003). These overlapping influences make it difficult to disentangle the specific contributions of play and to confirm that physical exploration itself is the causal factor.

### 2.3 Baby Steps to Causal Science

Recent studies have advanced the field toward testing causal relationships. Schröder et al. (2020) directly manipulated motor‐dependent object play in infants' homes. Over 8 weeks, the researchers randomly assigned eight‐month‐olds to either daily block‐play practice or an active “book‐reading” control condition. Infants who engaged in regular object manipulation showed improvements in visual form perception—measured by their ability to detect deviant shapes in an eye‐tracking task—relative to controls. Intriguingly, no comparable gains were found in approximate number sense acuity, suggesting that the effect was relatively specific to how infants learn to process object features through repeated hands‐on play.

Similarly, Veraksa et al. (2022) assigned 4–5‐year‐old children to one of three play interventions: role play, digital games, or board games (games with rules), over 7 weeks with two 20–30‐min sessions per week. Overall, play improved children's EF compared to a control group that received no play intervention. However, differences emerged based on play type: digital games affected all EF measures; board games improved all EF domains except behavioral inhibition (less so than digital games); and role play enhanced some EF aspects, namely visual working memory, shifting, and cognitive inhibition, but had less consistent effects on behavioral inhibition and auditory working memory, suggesting that play is beneficial for EF development, but different types of play have distinct developmental potentials, mechanisms, and benefits.

In the motor domain, Lobo and Galloway (2012) exposed 2‐month‐old infants to 3 weeks of enhanced handling and positioning experiences. Results showed both immediate advances in motor control, such as improved head control and sitting behaviors, and longer‐term improvements at 14 months in object transfer, creeping on hands and knees, and walking, supporting the idea that developmental outcomes arise from complex, experience‐dependent interactions between infants and their environment.

Along similar lines, Libertus et al. (2016) exposed 3‐month‐old infants to either active or passive motor training over 3 months. In the active condition, infants wore “sticky mittens” that facilitated grasping, while in the passive condition, infants wore mittens that did not support grasping. Results showed that infants who experienced active reaching training (i.e., “sticky mittens” group) at 3 months displayed increased object exploration 12 months later. Nevertheless, it is important to note that findings from the sticky mittens paradigm are mixed—with some studies reporting positive effects (e.g., Libertus et al. 2016; Needham et al. 2002) and others reporting null or modest effects (e.g., J. L. Williams et al. 2015)—indicating that more research is needed to clarify which factors drive these outcomes.

Together, these studies provide rare causal evidence that sensorimotor experience can directly shape aspects of early perception and exploration. However, they also indicate that other outcomes (e.g., domain‐general attention and numerosity, as seen in the Schröder et al.'s study) may require different types of or longer interventions. Similarly, the mixed findings from the sticky mittens paradigm further highlight the need to better understand the specific conditions and moderating factors that influence the effectiveness of such interventions and their impact on development (Van Den Berg and Gredebäck 2021).

## 3 Physical Play and Executive Functions

### 3.1 Movement as a Driver of Executive Functions

A particularly important frontier in the study of early cognition is the development of executive functions (EF), which include attentional control, working memory, inhibitory control, and cognitive flexibility. EF is often described as the set of top‐down processes that enable goal‐oriented behaviors, problem‐solving, and self‐regulation (Carlson et al. 2013; Zelazo and Carlson 2012). Yet, from an embodied cognition standpoint, EF is not merely a “brain‐based” skill controlling an otherwise passive body. Instead, EF emerges through repeated practice of *coordinated actions*—navigating physical challenges, engaging in goal‐directed play, using gestures to organize thought, and responding adaptively to environmental demands (Goldin‐Meadow and Alibali 2013; Keller et al. 2023; Lockman and Tamis‐LeMonda 2021; Rhoads et al. 2018; Rose et al. 2012, 2016; M. Wu et al. 2017).

Several lines of research—most of which have been conducted with children growing up in WEIRD (Western, Educated, Industrialized, Rich, Democratic; Henrich et al. 2010) societies—have highlighted the importance of early sensorimotor experience for the development of EF (Diamond and Ling 2020; Hudson et al. 2021). For example, infants who are early to reach basic motor milestones (like crawling or sitting upright) show different trajectories in attention shifting or inhibitory control compared to peers who attain similar milestones later (Campos et al. 2000; Iverson 2022). Other findings suggest that qualitative changes in motor ability—such as learning to stand or refining fine‐motor coordination—may help children practice the cognitive processes central to EF (Miquelote et al. 2012; Willoughby et al. 2021; M. Wu et al. 2017). By physically exploring their environments (e.g., pulling to stand, manipulating toys), infants and young children engage in goal‐directed actions that rely on core EF skills like sustained attention, planning, and inhibitory control. For instance, the development of manual actions is closely intertwined with visual attention, as infants use them to increase visual saliency and support sustained attention and object exploration during play (Franchak et al. 2024; Mendez et al. 2024; Yang et al. 2023).

In contrast, if a particular environment offers limited or overly passive modes of engagement, children may have fewer opportunities to exercise EF skills. Repeatedly watching a video that auto‐plays and does not require any interactive manipulation may offer minimal challenge to inhibitory control or cognitive flexibility (Paoletti et al. 2025; Barr et al. 2010). In line with this, Koşkulu et al. (2021) reported that the number and complexity of toys available during free play influence the duration, frequency, and quality of solo and joint attention.

Understanding the developing relations between the body, object affordances, and EF requires testing children's physical play as not just “exercise” for the body, but also as a core driver of developing cognitive systems because the body provides challenges on the fly—like a piece that doesn't fit, a tower that collapses, or a toy that rolls out of reach—that the child must learn to navigate using EF.

That said, most available findings on sensorimotor experiences and EF remain correlational, emphasizing the pressing need for controlled manipulations of children's everyday play contexts to clarify how specific motor affordances might directly shape EF. One could investigate whether repeated engagement in demanding object‐manipulation tasks—such as *systematically* fitting shapes into intricate puzzles—selectively enhances inhibitory control or working memory in infants and toddlers. Conversely, studies that temporarily remove key motor affordances could point to which aspects of motor learning prove most critical for EF development. Ultimately, ecologically valid intervention designs—particularly those that systematically adjust the difficulty or novelty of movement‐based activities—are poised to offer insights into how sensorimotor engagement actively scaffolds emerging cognitive control processes.

### 3.2 The Challenges in Testing Causality in the Wild

Why have so few studies tackled causal questions about children's physical play and EF in the real world? One primary obstacle is that play, by definition, is spontaneous, self‐directed, and context‐dependent (Lockman and Tamis‐LeMonda 2021; Tamis‐LeMonda and Bornstein 1991). Children may lose interest in contrived tasks, deviate from the instructions, or simply resist attempts at standardization. Parents, caregivers, and peers also shape the direction of play—actively or passively—and these social variables are difficult to control (Clackson et al. 2019; Tamis‐LeMonda and Schatz 2019; Wass and Goupil 2022).

Moreover, “physical experience” is not a single variable but rather a constellation of forces. The type of toy, the child's motor skills, the setting (floor vs. high chair vs. playground), the social climate, and individual temperament all interact. Attempting to isolate and manipulate just one dimension—such as offering only certain types of toys—may be overshadowed by broader influences, such as siblings sharing the toys or parents introducing alternative objects. Additionally, confounds abound in real‐world settings like homes or schools: differences in socio‐economic status, cultural norms around caregiving, availability of space, or caregiver schedules could all shape children's play experiences. The richness of naturalistic data, along with the confounding factors inherent to it, often requires advanced computational approaches to analyze them or a very large sample size to account for these factors. Those challenges are likely to hinder engagement with these types of experimental‐naturalistic designs.

Additionally, several key considerations for designing causal studies of naturalistic play have not been established, including design questions such as how long should the experimental manipulation last? How much daily play is necessary to produce measurable effects? Is the link between play and cognitive development direct, or does it follow a more complex, cascading pathway? Previous correlational and descriptive studies offer useful starting points (Babik et al. 2022; Karmazyn‐Raz and Smith 2023; Swirbul et al. 2022; Werchan et al. 2024). For example, the duration of children's play observed in correlational studies, along with the observed cognitive and behavioral changes during that time window, can serve as useful guidance for designing experimental interventions. Those findings should be expanded in future work.

Finally, measuring the outcomes of physical play can be tricky. While lab‐based tasks might capture certain aspects of EF or spatial cognition, these tasks often simplify or distort the child's natural behaviors. Assessing infant and preschool cognition in ecologically valid ways—perhaps through extended video coding of their spontaneous speech, problem‐solving, or social interactions—requires intense labor and methodological care (Wass and Jones 2023), especially if combined with neuroscientific methods (Gervain et al. 2023; Matusz et al. 2019; Noreika et al. 2020; Pinti et al. 2018). Moreover, the relationship between play and cognitive development is likely not linear or domain‐general; different types of play may affect distinct cognitive domains through varying developmental pathways. Capturing this complexity necessitates fine‐grained analytical techniques.

All of this complexity has led many researchers to rely on correlational designs or lab‐based protocols, where standardization is easier but ecological validity is lower. Nevertheless, we believe a shift in approach is needed to push the field toward a science of play‐based causation. Such a shift can be done through a scaffolded strategy, beginning with controlled laboratory experiments to establish causal links before attempting replications in naturalistic contexts. Such a stepwise progression not only strengthens the scientific foundation of the research but also ensures that efforts are efficient, ethical, and respectful of children's time and well‐being.

## 4 Proposals for New Research of Play‐Cognition Causality

### 4.1 Systematically Manipulating Object Affordances and Play Contexts

A powerful yet not fully harnessed strategy involves experimentally altering the types and affordances of toys or play materials children encounter in their daily lives while all other factors vary naturally. Consider a study where researchers provide one group of toddlers with toys that demand frequent manual exploration (e.g., shape sorters, building blocks, nesting cups) and another group with more passive, less manipulative toys (e.g., screens or simple plush dolls). Parents in each group could be guided to encourage or enable daily sessions of free play with the assigned toys, and researchers would track both immediate changes in play behaviors (e.g., the frequency of exploratory gestures, attention shifts, joint attention episodes) and longer‐term outcomes in EF tasks or word learning (Schröder et al. 2020).

In a similar manner, experimenters could switch the sets of toys midway through the study or systematically remove certain affordances (e.g., shapes that fit easily vs. shapes that require more trial‐and‐error). Video recordings, motion capture, or wearable eye‐trackers could document how children physically interact with these objects, revealing micro‐level patterns of exploration, attention, and possibly frustration or problem‐solving strategies (De Barbaro 2019; De Barbaro and Fausey 2022). By comparing the developmental trajectories of children who receive different types of play affordances—and by carefully measuring relevant cognitive endpoints—researchers could identify causal links between object‐based sensorimotor experiences and emerging EF.

While children's play experiences outside the lab will likely differ due to various natural factors beyond the study's control, these differences are expected to balance out across groups. Consider an experiment in which one group of children is provided with high‐affordance toys and a second group is provided with low‐affordance toys, and both groups are asked to play at school for 1 hour per day. Outside of that hour, some children may engage with tablets, while others may play with high‐affordance games. However, it is unlikely that these differences will be linked exclusively to one of the two groups; rather, they are more likely to vary across children. The key here is that the play experiences outside the lab for each group will likely be similar overall, allowing for the isolation of the specific effects of the experimental manipulation. The impact of such an intervention may be small due to uncontrollable factors outside the experiment, but this is precisely the point. The goal is not necessarily to identify a dramatic shift in development but to understand how small, cumulative experiences influence development over time.

Although overall group factors will likely vary across participants, potentially canceling each other out, attention must be given to the effects elicited by the experimental manipulation. In other words, it is important to identify noise that varies at random versus systematic noise.

For instance, in social interactions, caregivers or nursery teachers who observe children struggling with a shape sorter might intervene differently than when a child is playing with a digital tablet (Clackson et al. 2019). The scaffolding provided by an adult might be a key factor that either amplifies or diminishes the impact of the physical toy itself. Hence, a fuller experimental approach would also track and, if possible, standardize or train certain caregiver behaviors (e.g., prompting the child to try again, commenting on shapes, praising efforts) while still preserving the naturalistic flavor of the environment.

In addition to controlled interventions, cross‐cultural studies offer a complementary avenue for identifying causal relations. Variations in caregiving practices, play routines, and access to specific types of toys across cultural contexts serve as naturally occurring “experiments” that can provide insights into how these differences shape cognitive development. For instance, some cultures emphasize structured object play with educational toys, while others promote free exploration in tool‐rich environments (Gaskins et al. 2007; Lew‐Levy et al. 2022; Samuelsson 2020). However, such studies must contend with the fact that many variables—socioeconomic factors, language use, parenting norms—tend to co‐vary, making causal inferences more difficult (Rentzou et al. 2019; Schirmbeck et al. 2020). Still, when designed carefully, cross‐cultural research can offer valuable constraints and boundary conditions for generalizing findings across diverse developmental contexts.

### 4.2 Potential Interventions in Homes, Nurseries, and Schools

Implementing these ideas in real‐world contexts requires creative, flexible designs that align with families' and educators' practical realities (Hendry and Scerif 2023; Scerif et al. 2025). To take a causality approach, researchers should partner with multiple daycare centers to *actively* introduce a “play rotation” program, where certain classrooms receive sets of high‐affordance, manipulable toys for a few months, while others continue with their usual play routines. Meanwhile, experimenters would periodically film free‐play sessions, measure children's EF performance with gentle, game‐like tasks, and perhaps collect eye‐tracking or kinematic data from a subset of children whose parents consent to more in‐depth assessment.

Over a longer time frame (e.g., six months to a year), researchers can ascertain whether these structured manipulations of the play environment generate differences in children's cognitive outcomes compared to controls. Such an intervention design, while logistically complex, would yield stronger causal evidence than typical correlational studies.

An alternative approach could involve collaborating closely with families to provide a variety of toys designed to target specific cognitive skills (Schröder et al. 2020). For example, one group of children could receive toys that promote mental rotation and spatial reasoning, while another group could be given toys aimed at enhancing attention and perception. Parents would be guided to engage with their children using these toys at home for a designated period, and researchers could then assess the children's development. This would help establish a causal link between the type of play activity and changes in cognitive skills.

Similarly, researchers could evaluate children's daily play routines and introduce a targeted shift in play materials. Children who typically spend a lot of time with tablets could be encouraged to play with high‐affordance toys instead. This would allow researchers to observe how the multitude of factors associated with playtime directly impacts cognitive outcomes.

## 5 Leveraging Computational Advances in Real‐Life Settings

### 5.1 Harnessing Video, Kinematics, and Eye‐Tracking in Real‐Life Settings

Technological advances have made it increasingly feasible to collect detailed data on children's in‐home or in‐classroom play. High‐resolution video cameras can capture moment‐by‐moment changes in posture, hand movements, and object manipulations. Wearable eye‐trackers—miniature devices that sit on a child's head—can record where the child is looking as they explore objects, revealing attentional priorities in real time (Franchak et al. 2011; Franchak et al. 2024). Likewise, lightweight motion sensors or depth cameras can provide kinematic data on children's hand trajectories and body movements (Ossmy et al. 2021; Saha et al. 2024).

By applying these methods, researchers can build a rich, multidimensional view of how physical exploration unfolds (Xu et al. 2020). In the study comparing children's interactions with high‐affordance toys versus low‐affordance toys, investigators could point to differences in the *temporal structure* of play: Are children more likely to revisit the same object multiple times? Do they engage in more frequent posture changes or gaze shifts when using certain toys? Are there distinct patterns in how they coordinate manual actions and visual attention—perhaps scanning a puzzle piece before attempting to fit it, or looking back at a caregiver for cues?

These micro‐level data can be aligned with standardized measures of attention, memory, language, or EF collected periodically throughout the study (Perapoch Amadó et al. 2025; Werchan et al. 2024; White et al. 2021; Yu and Smith 2017). If findings show that children with greater access to high‐affordance toys develop more varied kinematic patterns and show better inhibitory control or problem‐solving later on, stronger inferences can be drawn about how physically engaging play fosters cognitive growth. Moreover, advanced analytic tools—such as dynamic systems modeling or hidden Markov models—can help identify emergent structures in children's interactions that might be overlooked in simpler coding schemes (Le et al. 2021; Lichtenberg et al. 2024; Stifter and Rovine 2015).

### 5.2 Integrating Embodied Computational Models

One innovative approach to circumvent some challenges of real‐world experimentation is to build neurorobotic or computational models that mimic key features of children's sensorimotor exploration (Cangelosi and Schlesinger 2018; Ossmy et al. 2018, 2024; Oudeyer 2017). In these simulations, artificial agents with biologically inspired neural networks and motor repertoires can be placed in virtual or physical environments containing different objects (Oudeyer 2017). Researchers can then systematically alter the agent's motor abilities, the complexity of objects, or the types of social feedback the agent receives. Changes in the agent's “cognitive” states or how its representational structures develop over time, inform which patterns of physical exploration lead to the most robust learning.

Although such models are abstractions, they offer a test bed for pinpointing mechanistic principles—e.g., does having more varied motor actions accelerate category learning or does it distract from forming stable representations? Do repeated sensorimotor patterns help the agent attend to crucial features, or do they trap it in a limited loop of behaviors? Once identified, these principles can be tested in real‐life studies. If a model suggests that partial constraints on an agent's movement (akin to the “sticky mittens” paradigm; Needham et al. 2002) accelerate object category formation, researchers can devise a parallel intervention with human infants to see whether the same effect emerges.

## 6 Broader Implications for Developmental Science and Intervention

Establishing causal links between physical play and cognitive development is not a purely academic exercise. Insights gleaned from these studies could powerfully inform the design of educational materials, early interventions, and public health guidelines. If some toys that demand consistent manual exploration and problem‐solving prove to enhance EF skills, policy‐makers and educators could prioritize ensuring that preschools (especially in underserved communities) are equipped with such materials. For example, a recent study conducted in low‐income settings in South Africa and The Gambia challenges the idea that children from low‐income countries have poorer EF than those from high‐income countries solely due to lower socioeconomic status (Milosavljevic et al. 2024). However, what factors predict EF in this context? The quality of playtime may serve as a resilient factor in high‐ and low‐income groups, but further research is needed to confirm its role. Similarly, if certain sensorimotor routines in infancy reliably accelerate language or spatial cognition, pediatricians and early childhood specialists might incorporate these findings into parenting advice.

Moreover, while infancy and early childhood are prime periods for investigating how the body shapes cognition, the same principles likely apply to older children, adolescents, and adults, albeit in different forms. As R. Wu and Strickland‐Hughes (2019) argues, humans continue to learn new motor‐based skills and adapt to novel technologies throughout their lives. Even older adults, widely assumed to rely on top‐down knowledge, may benefit from physically interactive learning environments (R. Wu et al. 2016). Observational data suggest that cognitively stimulating physical activities—like dance classes, sports, or complex manual crafts—are associated with better cognitive functions in older populations (Marmeleira and Duarte Santos 2019) or children with brain lesions (Houser et al. 2024; Kang et al. 2011; Khaleqi‐Sohi et al. 2022).

However, because real‐world interventions are rarely randomized or experimentally manipulated, we have minimal causal evidence. If an older adult regularly engages in dance, do we know that the motor aspects of dance specifically bolster executive control, or might it be the social and emotional stimulation of the group class? Do the skill demands—like memorizing choreography—uniquely strengthen working memory (Cortese and Rossi‐Arnaud 2010)? Or do self‐selection biases, in which more cognitively healthy adults choose to dance, explain the correlation (Noguera et al. 2020)? Answering such questions would again require carefully designed manipulations of physical experiences and tracking subsequent changes in cognitive functioning (Nastase et al. 2020). It may also help us understand how best to harness emerging technologies (like virtual reality or robotics) to deliver beneficial sensorimotor challenges that promote cognitive resilience across the lifespan.

## 7 Conclusions

The emerging shift to naturalistic infancy research is important and widely welcomed but has, until now, been largely descriptive. This observational phase has likely been a necessary step in understanding both the strengths and limitations of the cutting‐edge methodologies at researchers' disposal, as well as the quality and level of detail they capture in naturalistic settings. This extensive body of descriptive research has been invaluable in refining key variables and calibrating the granularity of analyses. It also provides a critical foundation for designing high‐quality causal studies of play, drawing on existing correlations between the amount and type of play and cognitive outcomes.

With a clearer understanding of what can be measured and the quality of the available data, the next step is to move beyond mere observation—integrating these variables into formal models, developing and testing theories, and applying the rigorous scientific methodologies that have long driven progress in our field. This causal science of early play is essential for understanding *when* and *how* play influences and contributes to developmental cognitive outcomes across cultures.

## Author Contributions

**Giulia Serino:** writing – original draft, writing – review and editing. **Ori Ossmy:** conceptualization, writing – original draft, funding acquisition, writing – review and editing.

## Conflicts of Interest

The authors declare no conflicts of interest.

## Data Availability

Data sharing not applicable to this article as no datasets were generated or analyzed during the current study.
